# Role of Indole Scaffolds as Pharmacophores in the Development of Anti-Lung Cancer Agents

**DOI:** 10.3390/molecules25071615

**Published:** 2020-04-01

**Authors:** Jyothi Dhuguru, Rachid Skouta

**Affiliations:** Department of Biology, University of Massachusetts, Amherst, MA 01003, USA; jyothi.dhuguru@gmail.com

**Keywords:** lung cancer, indole, non-small cell lung cancer, kinase inhibition, apoptosis, DNA topoisomerase, histone deacetylase inhibition

## Abstract

Lung cancer is the leading cause of death in men and women worldwide, affecting millions of people. Between the two types of lung cancers, non-small cell lung cancer (NSCLC) is more common than small cell lung cancer (SCLC). Besides surgery and radiotherapy, chemotherapy is the most important method of treatment for lung cancer. Indole scaffold is considered one of the most privileged scaffolds in heterocyclic chemistry. Indole may serve as an effective probe for the development of new drug candidates against challenging diseases, including lung cancer. In this review, we will focus on discussing the existing indole based pharmacophores in the clinical and pre-clinical stages of development against lung cancer, along with the synthesis of some of the selected anti-lung cancer drugs. Moreover, the basic mechanism of action underlying indole based anti-lung cancer treatment, such as protein kinase inhibition, histone deacetylase inhibition, DNA topoisomerase inhibition, and tubulin inhibition will also be discussed.

## 1. Introduction

Lung cancer remains the deadliest disease on record, with approximately 2.09 million new cases reported each year. According to the World Health Organization, it is responsible for 1.7 million cancer deaths worldwide per year [1]. There are two major types of lung cancer: small cell lung cancer (SCLC) and non-small cell lung cancer (NSCLC). Among these two types of lung cancers, NSCLC is responsible for approximately 80%–90% of cancerous tumors [2,3]. NSCLC is further divided into three major histological subtypes: squamous-cell carcinoma, adenocarcinoma, and large-cell carcinoma. SCLC and squamous-cell carcinoma are strongly linked to smoking, while adenocarcinoma is the most common type of lung cancer in patients who have never smoked [4].

Treatment of lung cancer is commonly addressed by surgical removal of the affected area and by chemotherapy, which applies a combination of one or more drugs in conjunction with radiation therapy. However, multidrug resistance and the severe side effects associated with chemotherapy persists as one of the major challenges in the treatment of lung cancer.

### 1.1. Indole and its Pharmacological Significance

**Figure 1:**
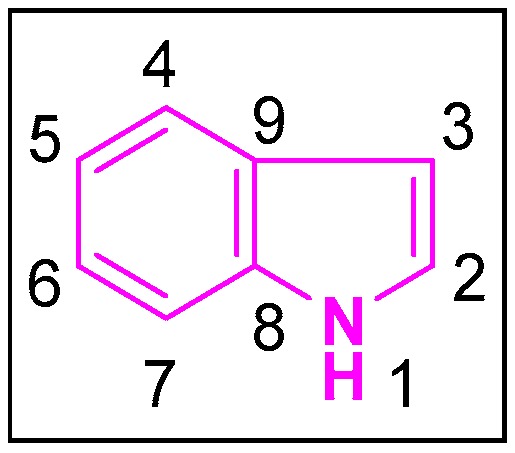
Structure of indole.

Indole is a pervasive and naturally occurring heterocyclic compound, the structure of which comprises of a six-membered benzene ring tethered to a five-membered pyrrole ring [5] (Figure 1). With its 10 π-electrons, indole follows Huckel’s rule of aromaticity. It is more prone to electrophilic substitution reactions similar to the benzene ring. Molecular orbital studies showed that the 3-position of indole is more reactive towards the electrophilic substitution reactions. Indoles can undergo N-substitution reactions under basic conditions owing to the slightly acidic nature of the—NH bond in indole [6]. The first synthesis of indole was accomplished in 1866 by Adolf von Baeyer [7]. Due to the diverse applications of the indole scaffold in both the biological and pharmacological fields, hundreds of synthetic schemes were published. The most prominent and widely used ones are those reported by Fisher [8], Bischler [9], Hemetsberger [10], Nenitzescu [11], Bartoli [12], and others [13,14].

Owing to its bioavailability and pharmacological applications, indole is considered the most privileged scaffold in heterocyclic chemistry with such widespread applications as antiviral, antimicrobial, anti-inflammatory, anti-cancer, anti-hypertensive, anti-diabetic, and antioxidant [15,16,17].

### 1.2. Natural Abundance of Indoles 

Naturally occurring indole derivatives, such as glucobrassicin and indole-3-carbinol, are commonly found in cruciferous vegetables like cabbage, broccoli, and sprouts [18]. Indole-3-carbinol and its dimeric product 3,3′-diindolylmethane have been found to induce apoptosis in cancer cells [18]. This is a property that has increasingly commanded the attention of researchers from around the world. To get the greatest benefits, indole rich sources belonging to the Brassica family are particularly recommended by the National Cancer Institute to reduce the risk of cancer [18]. 

**Figure 2:**
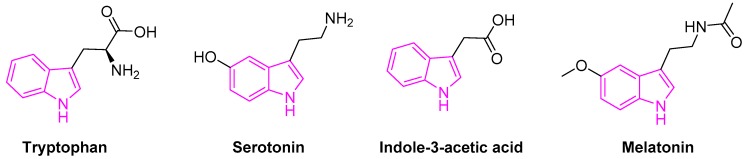
Indole scaffold in biologically important compounds.

Tryptophan containing indole is an essential amino acid found in many natural resources. It is also a biosynthetic precursor for the tryptamine, 5-hydroxy-tryptophan, an immediate precursor of serotonin (Figure 2). 

Serotonin (5-hydroxytryptamine) is an important neurotransmitter associated with the transmission of nerve impulses. It further serves as a precursor to melatonin, a neuro-hormone secreted by the pineal gland. Besides, melatonin is involved in several physiological processes beyond the circadian rhythm [19,20]. Indole-3-acetic acid is the most common and widely studied plant hormones of the auxin class (Figure 2) [21]. It is also the normal urinary tryptophan metabolite observed to be elevated in patients with various forms of liver disease [22].

### 1.3. Significance of Indole Based Drugs

**Figure 3:**
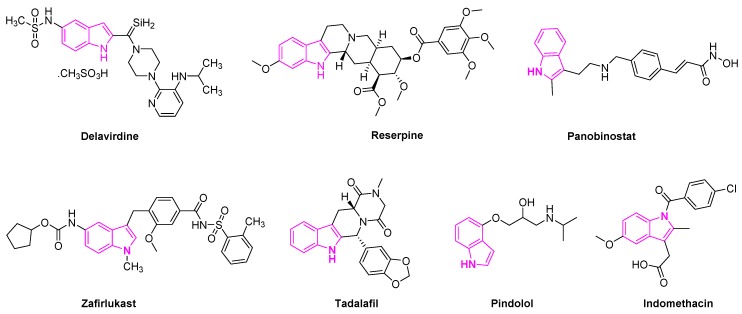
Indole scaffold in Food and Drug Administration (FDA) approved drugs.

Delavirdine is an indole based anti-retroviral drug approved by the Food and Drug Administration (FDA) in 1997 for the treatment of HIV patients (Figure 3). The drug acts as a non-nucleoside reverse transcriptase inhibitor and an inhibitor of the cytochrome P450 enzyme CYP3A4 [23]. Reserpine is a known indole alkaloid derived from the *Rauwolfia serpentina* plant. It has been utilized as an anti-psychotic and anti-hypertensive agent to treat high blood pressure [24]. Panobinostat is an oral histone deacetylase inhibitor developed by Novartis and approved to treat multiple myeloma. The drug is currently in phase II trials to treat acute myeloid leukemia [25] and phase I and II clinical trials for the treatment of AIDS [26]. Zafirlukast is the first orally active indole-like leukotriene-receptor antagonist approved by the FDA to treat asthma (Figure 3) [27]. It is mostly used in combination with an inhaled steroid and/or long-acting bronchodilator. Tadalafil is another indole based drug approved for the treatment of erectile dysfunction in men [28]. Pindolol is an antagonist of the serotonin 5-HT1A receptor and it is used to treat hypertension and as an anti-depression agent [29,30]. Indomethacin is a non-steroidal anti-inflammatory drug applied to the treatment of severe osteoarthritis, rheumatoid arthritis, gouty arthritis, and ankylosing spondylitis [31] (Figure 3).

### 1.4. Methodology for Bibliographic Search

We used the following databases to survey the literature reporting indole scaffold usages for lung cancer in in vitro and in vivo models: 

1. Scifinder website at https://scifinder.cas.org/.

2. PubMed website at https://www.ncbi.nlm.nih.gov/pubmed/.

In addition, we filtered the available articles based on the following criteria:

a. Articles must have chemical structures of indoles.

b. Articles must have biology data of indoles.

c. We included articles with bioactivity data of indoles specifically in lung cancer.

d. We excluded articles with bioactivity data of indoles other than lung cancer. 

## 2. Indole Derivatives as Anti-Lung Cancer Agents

**Figure 4:**
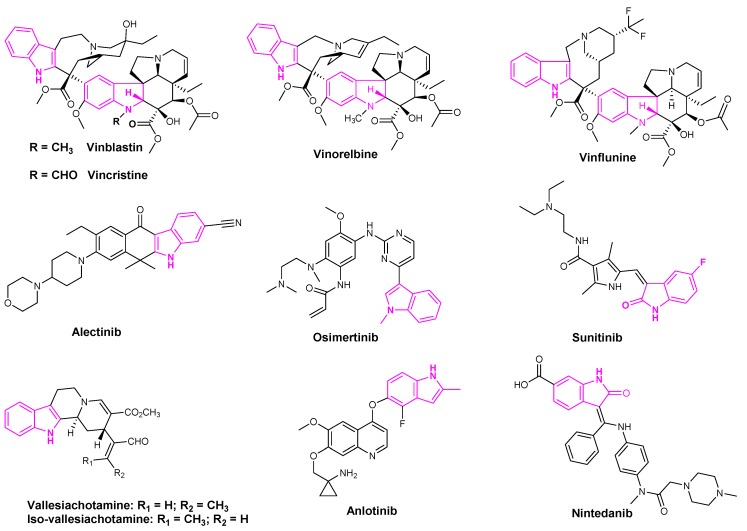
Indole containing anti-lung cancer drugs in clinical and pre-clinical stages.

Indole based alkaloids share great historical popularity as anti-cancer drugs. The most prominent among them have been vincristine, vinblastine, vinflunine, and vinorelbine derived from the plant *Catharanthus roseus* [32] (Figure 4). With over 150 alkaloids, *C. roseus* is the most well-studied medicinal plant. Vinorelbine is the most commonly used drug to treat lung cancer and vinblastine in combination with cisplatin is used in the treatment of NSCLC [33]. The anti-cancer activity of these derivatives was attributed to their ability to dissolve the mitotic spindles and cell division resulting from the microtubule disruption [33].

Alectinib is a popular indole based oral drug developed by AstraZeneca. FDA approved the drug in 2015 for the treatment of crizotinib-resistant NSCLC [34]. Sunitinib is a multi-targeted receptor tyrosine kinase inhibitor that was approved in the first-line treatment of advanced renal cell carcinoma and gastrointestinal stromal tumors [35]. Osimertinib was also approved by the FDA in 2015 to treat metastatic EGFRT790M mutation-positive NSCLC patients [36]. Osimertinib and sunitinib are the indole based drugs, marketed for the treatment of NSCLC, advanced renal cell carcinoma, and gastrointestinal stromal tumors, respectively [36] (Figure 4). Mishra et al. recently isolated seven indole-based alkaloids and studied the anti-proliferative activities of their isolated compounds. They observed that aglycone indole based alkaloids, namely vallesiachotamine and iso-vallesiachotamine, exhibit anti-tumor activity with The half maximal inhibitory concentration (IC_50_) values of 4.24 μM and 3.79 μM, respectively, against H1299 human lung cancer cells (Figure 4) [37]. Anlotinib is a novel oral multi-target tyrosine kinase inhibitor, used for the third-line treatment of advanced lung cancer [38]. 

**Figure 5:**
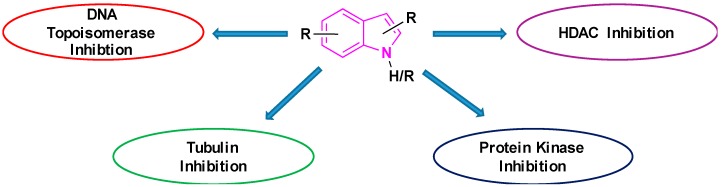
Basic mechanism of action for indole containing anti-lung cancer drugs.

The potency of indoles as anti-cancer agents is exemplified by (i) various indole derivatives, such as indole-3-carbinol, indole-3-carboxaldehyde; (ii) functionalized indoles, such as diaryl-indoles, indolyl chalcones, indolyl azoles, and (iii) bisindole derivatives [39]. Several studies cast light on the anti-cancer efficacy of indole derivatives [40,41,42,43]. Besides, several reviews were submitted in recent years exploring the biological and pharmacological applications of indole derivatives as anticancer agents [39,44,45,46,47]. Though many comprehensive reviews were published, focusing on indole derivatives as anti-cancer agents, so far to our knowledge, there are no reports specifically focused on the efficacy of indole-based compounds in combatting lung cancer. Therefore, we are seeking to fill this gap by presenting a brief review focused on the development of indole-based derivatives as anti-lung cancer agents. In this paper, we included work between 2000 and 2019, of indole-based derivatives in the clinical and subclinical stages of development to combat lung cancer. As it is not possible to include the synthesis of all indole based anti-lung cancer agents, we are reporting herein the synthesis of some of the selected derivatives. We apologize in advance for possible neglect of any relevant work including synthesis on the subject. Basic mechanisms by which indole derivatives exhibit anti-lung cancer properties involve the induction of apoptosis, microtubule inhibition, protein kinase inhibition, histone deacetylase inhibition, and DNA topoisomerase inhibition (Figure 5). In the following sections, we will discuss several derivatives of indoles, developed and understudy for their prospective use against lung cancer, along with their mechanism and synthesis of some of the selected derivatives. 

### 2.1. Indole Derivatives as Kinase Inhibitors

Protein tyrosine kinases are a large multi-gene family comprising key signaling molecules and, therefore, are attractive drug targets. There are two types of tyrosine kinases: (a) receptor kinases, (b) non-receptor kinases. Receptor kinases comprising transmembrane, an extracellular domain, and an intracellular domain. Non-receptor kinases comprise only an intracellular domain with no extracellular or transmembrane. Both kinases play critical roles in cellular signaling, cell growth, cell division, cellular metabolism, and apoptosis. Studies have confirmed that any kind of upregulation or overexpression of the receptors, or any mutation in these receptors, can lead to lung cancer [48,49,50]. Therefore, indole based small molecule inhibitors targeting the kinase receptors have become a promising option for drug discovery scientists in their attempt to battle lung cancer. 

### 2.2. Targeting Akt Signaling Pathway

Nearly 51% of the NSCLC patient samples and 74% of the NSCLC cell lines show activation of phosphatidylinositol 3-kinase/Akt/mTOR pathway [51,52]. Protein kinase B/Akt regulates several key cellular processes such as cell division, cell proliferation, angiogenesis, and cell survival. It activates signaling pathways downstream of tyrosine kinases and dysregulation of these signaling pathways has been observed in several human cancers. Because of the biological roles of Akt signaling pathways, several categories of inhibitors targeting these kinases were developed for NSCLC treatment [53,54].

Nesi et al. designed and synthesized a novel set of 2-oxindole-derivatives ((**6a–c**), (OXIDs)) targeting serine/threonine kinases, 3-phosphoinositide-dependent protein kinase-1 and/or Akt pathways [55]. They revealed the significance of the oxindole core along with the thiophene substituent, in the 3rd position in exerting the antitumor activity. In their studies, they found that the compounds **6a-c** showed six-fold higher antitumor activity than Akt inhibitor perifosine and were more potent than sunitinib with 2.5 to 10-fold higher anti-cancer activity. IC_50_ values ranged from submicromolar to micromolar. These studies revealed the potential of oxindole core in the further development of future therapeutic agents to treat NSCLC [55]. 

**Scheme 1:**
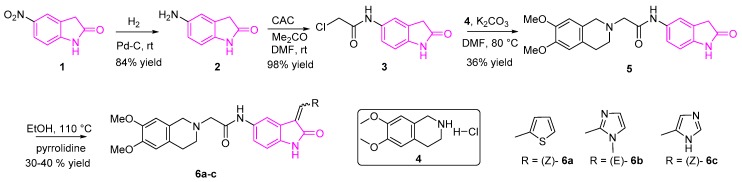
Synthesis of 2-oxindole-derivatives (OXIDs) (**6a–c**) adapted from the reference [55].

The synthesis of OXIDs is depicted in Scheme 1 [55]. Briefly, compound **2** (5-amino-2-oxindole) was synthesized by the Palladium on carbon (Pd/C) catalyzed hydrogenation of compound **1,** followed by condensation with the 2-chloro-acetylchloride resulting in compound **3**. The subsequent reaction of intermediate **3** with compound **4** in the presence of potassium carbonate afforded derivative **5,** followed by the Knoevenagel reaction involving the corresponding heterocycles resulting in the desired OXIDs compounds (**6a–c**). 

### 2.3. Epidermal Growth Factor (EGFR) Inhibition

**Scheme 2:**
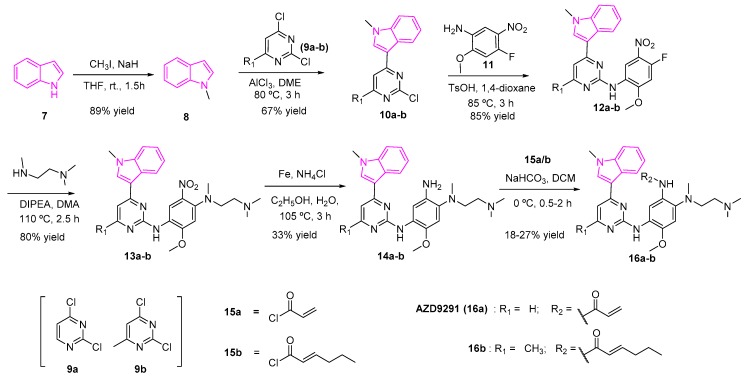
Synthesis of compounds **16a and 16b** adapted from the references [56,57].

**Figure 6:**
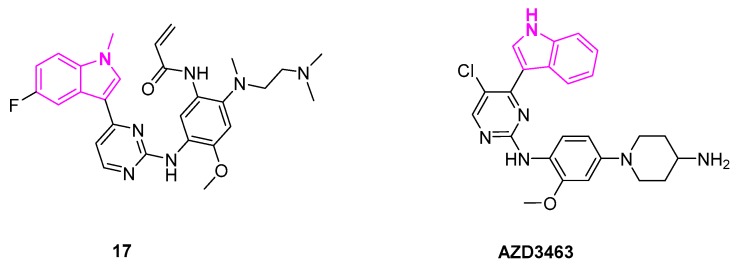
Structures of compounds **17** and **AZD3463**.

Osimertinib (AZD9291, **16a**) is an FDA-approved drug to treat non-small lung cancer as an EGFR inhibitor [36]. However, AZD9291 showed low specificity to EGFR along with severe side effects and cardiotoxicity at high doses (Scheme 2). A structure-activity relationship study of AZD9291 reported by Zhao et al. showed that the fluorinated analog (**17**, Figure 6) improved selectivity towards EGFRT^790M/L858R^ double mutants with IC_50_ as low as 8 nM and a 200-fold increase in the selectivity towards wild type EGFR. It was theorized that the fluorinated indole (**17**) plays an important role in enhancing the binding interaction with T790M active binding domain of EGFR and reducing the demethylation ability of the indole ring, which in turn, can enhance the anti-metabolism ability of AZD9291 derivatives. The fluorinated derivative of AZD9291 (**17**), however, resulted in the loss of kinase activity. Next, the authors designed compound **16b** by the aid of molecular docking studies to obtain an optimized derivative without fluorine substitution. They showed that **16b** can serve as a potent EGFRT^790M/L858R^ inhibitor [56]. The synthesis of compounds **16a** and **16b** is shown in Scheme 2 [56,57]. 

The first step of the synthesis involved the methylation of indole (**7**) using methyl iodide and sodium hydride to furnish the methylated indole (**8**). A mixture of the 2,4-dichloro-pyrimidine (**9a**) or 2,4-dichloro-6-methyl-pyrimidine (**9b**) in dimethoxyethane and AlCl_3_ with 1-methylindole (**8**) yielded compound **10a** or **10b** respectively. A nucleophilic aromatic substitution reaction between the amino compound **11** and the chloro pyrimidine **10a/10b** in generated the fluoro intermediate (**12a/12b**). The reaction of the fluoro compounds (**12a/12b**) with N,N,N-trimethylethane-l,2-diamine in diisopropylethylamine and dimethylacetamide gave the nitro intermediates **13a/13b,** which was subsequently reduced to the amines **14a/14b**. The latter amines were treated with acryloyl chloride to afford the desired final products (**16a**/**16b**) (Scheme 2).

### 2.4. Anaplastic Lymphoma Kinase Inhibition

Anaplastic lymphoma kinase (ALK) belongs to the family of tyrosine kinases and is regarded as a potential target for the treatment of neuroblastomas as ALK gene mutations and overexpression is frequently observed in neuroblastoma patients. **AZD3463** is an ALK inhibitor designed by Astrazeneca to overcome the acquired resistance towards crizotinib (Figure 6). Crizotinib is an FDA-approved drug for the treatment of NSCLC [58]. It is further known to be a potent second-generation ALK (anaplastic lymphoma kinase) inhibitor, which proved effective against a broad spectrum of ALK rearrangements and ALK mutations [59].

**Scheme 3:**
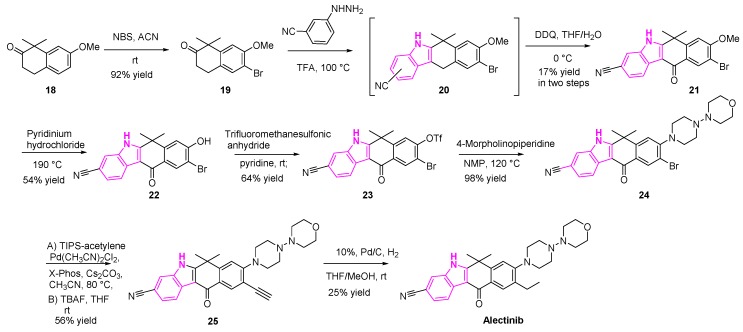
Synthesis of alectinib adapted from the references [60,61].

Alectinib, as mentioned earlier, is an FDA approved drug for the treatment of NSCLC (Scheme 3). It is a highly selective and potent ALK inhibitor. Synthesis of alectinib was accomplished in eight steps, as shown in Scheme 3 [60,61].

The bromination of compound **18** with *N*-bromosuccinimide, followed by the Fischer indole synthesis, resulted in the regioisomeric mixture (1:1, 1-CN- and 3-CN-derivatives) of compound **20**. The desired isomer was isolated and the 2,3-Dichloro-5,6-dicyano-1,4-benzoquinone (DDQ) oxidation was conducted, resulting in the ketone **21**. Demethylation of compound 21 with pyridinium hydrochloride generated the alcohol (**22**). The resulting alcohol (**22**) was then triflated and then reacted with 4-morpholinopiperidine to provide the bromo compound (**24**). The bromo intermediate (**24**) was then subjected to Sonogashira coupling with acetylene, followed by hydrogenation of the terminal alkyne generated the desired compound (alectinib), as shown in Scheme 3.

### 2.5. Protein Kinase C Inhibition

Protein kinase C (PKC) is a family of structurally related serine-threonine kinases that can be activated by G-protein coupled receptors [62]. PKC is implicated in several cellular functions and the regulation of survival signals in various cell types and, therefore, PKC has become a drug target for the development of anti-cancer drugs. The activation of PKC by various oncogenes can lead to the malignant transformation and abnormal expression of PKC isoforms, as detected in the head, neck, and other types of cancers. 

**Scheme 4:**
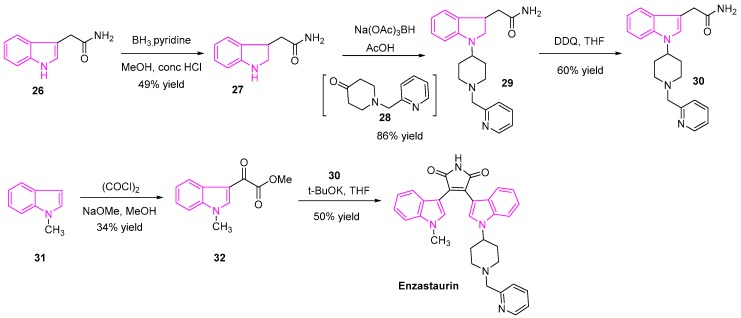
Synthesis of enzastaurin adapted from the reference [65].

Enzastaurin is a popular anti-tumor agent, which showed promising results with T-cell and B-cell lymphoma, multiple myeloma and other solid tumor malignancies (Scheme 4). Enzastaurin is a potent inhibitor of PKC and PI3K/AKT pathway. It is found to effectively inhibit tumor cell growth in both in vitro and mouse xenograft models [63]. Nakajima and his team showed that enzastaurin displayed cell growth inhibition with IC_50_ of 3–10 µmol/L, as exhibited in a panel of both SCLC and NSCLC cell lines [64,65]. Besides, they found that enzastaurin can suppress tumor-induced angiogenesis in A549 NSCLC xenografts [66]. The drug is undergoing clinical trials to evaluate its efficacy in the treatment of NSCLC and several other types of cancers [67]. 

The synthesis of enzastaurin is shown in Scheme 4 [65]. Briefly, the reduction of indole-3-acetamide (**26**) with BH_3_.pyridine gave the indolin-3-acetamide (**27**) followed by reductive amination with the ketone (**28**) resulted in the intermediate **29**. Compound **30** was obtained by oxidation of compound **29** using DDQ. On the other hand, the indole-3-glyoxylate ester (**32**) was synthesized from 1-methylindole (**31**) and oxalyl chloride. Finally, condensation of the amide intermediate (**30**) with indole-3-glyoxylate ester (**32**) in the presence of a base generated enzastaurin [67] (Scheme 4).

### 2.6. GSK Inhibition

**Scheme 5:**
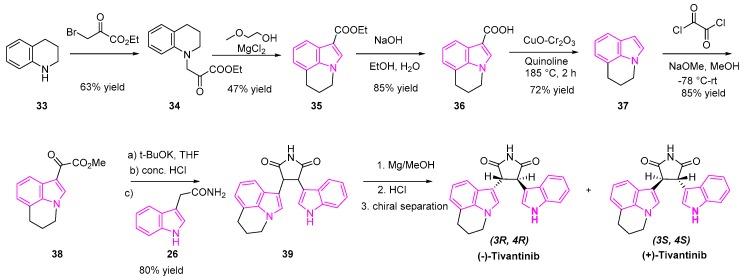
Synthesis of tivantinib adapted from the references [69,70].

Tivantinib is an oral, selective indole based small molecule MET inhibitor, being evaluated in advanced clinical trials for the treatment of NSCLC (Scheme 5). Early reports showed that tivantinib is a potent and highly selective inhibitor of receptor tyrosine kinase (cMET). However, further investigation led by Rix et al. revealed glycogen synthetase kinase (GSK)3α and GSK3β to be novel tivantinib targets. In their detailed study of mechanism, they found that tivantinib acts by the simultaneous inhibition of GSK3α and GSK3β leading to apoptosis in lung cancer cells [68]. Their experiments demonstrated that (−)-tivantinib was more potent than (+)-tivantinib in the inhibition of GSK3α and GSK3β. The latest investigation by Scagliotti et al. has shown that a combination of tivantinib and erlotinib can enhance treatment efficacy in patients with previously treated, EGFR mutant, non-squamous NSCLC, as compared to erlotinib alone [69].

The synthesis of tivantinib is shown in Scheme 5 [69,70]. The reaction of the tetrahydroquinoline (**33**) with the bromo-ethylpyruvate yielded the ketoester **34**, which was transformed to the ester indole **35** under anhydrous magnesium chloride treatment. Ester **35** was hydrolyzed to carboxylic acid intermediate **36** followed by decarboxylation reaction using copper chromite and quinoline afforded the indole **37**, which was treated with oxalyl chloride and methanol to obtain the ketoester **38**. Condensation of the ketoester **38** and indole-3-acetamide (**26**) generated the intermediate **39** as a racemic mixture of tivantinib. Preparative chiral HPLC purification yielded two optically pure diastereoisomers of (3R, 4R) (−)-tivantinib and (3S, 4S) (+)-tivantinib (Scheme 5).

### 2.7. Inhibition of Multiple Receptor Kinases

Anlotinib is a novel inhibitor targeting multiple receptor tyrosine kinases, namely vascular endothelial growth factor receptor type 2 and 3 (VEGF 2 and 3), the platelet-derived growth factor b (PDGFb), and the fibroblast growth factor 2 (FGF-2) [71] (Figure 4). Studies revealed that VEGF is a critical growth factor in tumor angiogenesis. Along with PDGF and FGF-2, it stimulates proliferation, migration, survival, and new vessel formation of endothelial cells in tumors. Studies showed that inhibition of these angiogenic receptor tyrosine kinases can be very effective in the treatment of tumors. Notably, the inhibitory effect of anlotinib was much more pronounced than with sunitinib, sorafenib, and nintedanib suggesting it as a potent anti-lung cancer drug. Anlotinib has emerged as a potential third-line treatment in advanced NSCLC patients [72]. 

Nintedanib is an oral, small molecule multiple kinase inhibitor (Figure 4). This drug in combination with docetaxel was approved worldwide for the treatment of NSCLC [73]. It functions as a tyrosine kinase inhibitor targeting three angiokinase inhibitors including VEGF (1–3), PDGF receptors (α/β) and fibroblast growth factors (1–3). Both in vitro and in vivo studies in human NSCLC xenografts have confirmed that by the inhibition of both AKT and mitogen-activated protein kinases signaling pathways, nintedanib resulted in the inhibition of cell proliferation, apoptosis in endothelial cells, and the cell types involved in angiogenesis [74]. 

### 2.8. Tubulin Inhibition

Tubulin is a globular protein comprising α and β-heterodimers, which polymerize to form the biopolymers of microtubules. Being the third major component of the cytoskeleton, microtubules play a critical role in cell division, in addition to aiding in both inter and intra-cellular movement and maintaining cell shape [75]. As microtubules play a key role in cell division, they are attractive targets for the development of anti-cancer agents. 

**Figure 7:**
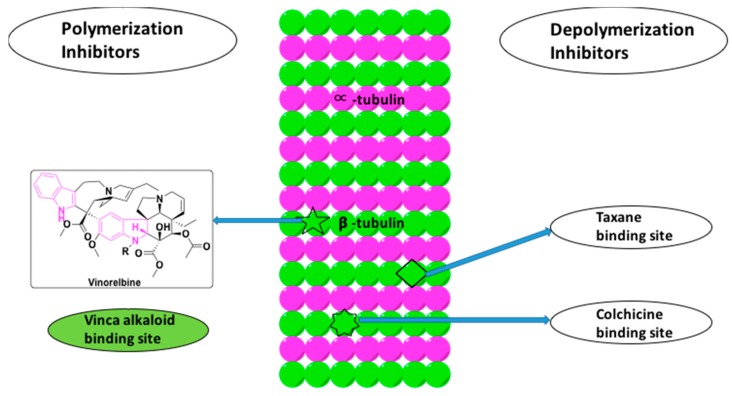
Binding sites in tubulin.

Tubulin inhibitors interfere with the polymerization and depolymerization of the microtubules. They are categorized as either tubulin stabilizing agents or destabilizing agents (Figure 7). While the tubulin stabilizing agents ultimately lead to the polymerization of microtubules by inhibiting depolymerization, the destabilizing agents function by inhibiting polymerization, thereby shortening the microtubules [76]. The indole based alkaloids and their derivatives, such as vincristine, vinblastine, vinorelbine, vinflunine, vindesine belong to the class of polymerization inhibitors that bind to the β-subunit of tubulin at a distinct region called as the vinca-binding site, as shown in Figure 7 [76]. 

Vinorelbine is a semi-synthetic indole based alkaloid extracted from *C. roseus* (Figure 7). It is a known microtubule inhibitor with selectivity towards mitotic microtubules. It disrupts the mitotic spindle formation and ultimately prevents cell division. Vinorelbine is used in combination chemotherapy with cisplatin and cetuximab to treat NSCLC [77]. Besides vinorelbine, vinflunine was also shown to have the potential to treat the NSCLC in the adjuvant chemotherapy with platinum-based drugs (Figure 4) [78]. Among the other counterparts, such as vincristine, vinblastine, and vinorelbine, vinflunine is found to have higher activities in vivo with low drug resistance. Therefore, vinflunine has evolved to be a more potent drug in lung cancer treatment [33].

**Scheme 6:**
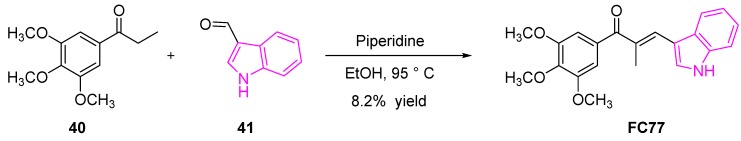
Synthesis of FC77 adapted from the reference [79].

Cong et al. reported an indole-chalcone derivative (**FC77**, Scheme 6) that can arrest cancer cell growth by binding to tubulin, exhibiting a similar mechanism of action as colchicine. **FC77** exhibited a potent growth inhibitor of a majority of NCI-60 human cancer cell lines with GI50 ~ 6 μM [79]. The ability of **FC77** to retain its cytotoxicity against multi-drug resistant cancer cell lines (with nanomolar GI values) and low cytotoxicity levels towards normal mobilized peripheral blood cells revealed that **FC77** may be a potential therapeutic agent in treating multi-drug resistant cancers such as NSCLC. Moreover, the direct interaction of **FC77** with tubulin and inhibition of microtubule dynamics with IC_50_ values of 3 nM compared to popular drugs such as paclitaxel and vincristine with IC_50_ values of 14 nM and 37 nM, respectively, makes it an attractive microtubule-targeting agent for the treatment of multidrug-resistant cancers [79]. 

The synthesis of **FC77** was accomplished in a single step, as shown in Scheme 6 [79]. The condensation reaction between the acetophenone derivative (**40**) with the indole-3-carboxaldehyde (**41**) in the presence of piperidine afforded the target compound (**FC77**). 

**Scheme 7:**
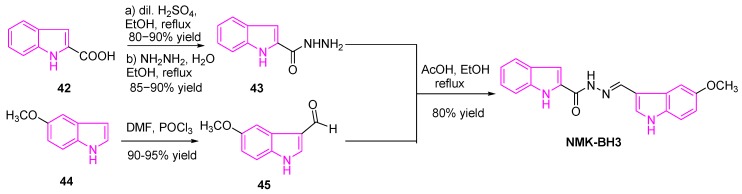
Synthesis of NMK-BH3 adapted from the reference [80].

Das et al. recently synthesized a series of six bis(indolyl)-hydrazide−hydrazones (NMK-BH compounds), in order to develop novel and efficient microtubule-targeting chemotherapeutic agents against lung cancer. They achieved this by linking the two indole rings with an active pharmacophore linker, as shown in Scheme 7. Cytotoxicity studies of these compounds against human lung adenocarcinoma A-549 cells revealed that **NMK-BH3** with the electron-donating 5-OMe group and the indole rings tethered to the C-2 position of the indole-A was found to be more potent than other combinations. The mechanism of action of **NMK-BH3** in NSCLC (A549 cells) revealed the disruption of the mitotic spindle and depolymerization of the interphase microtubule network ultimately led to apoptotic cell death. This is signified by the overexpression of Bax, p53, caspase 3, and caspase 9 and decrease in the Bcl-2 expression [80]. Their experiments revealed that **NMK-BH3** was more sensitive to human lung adenocarcinoma A549 cells with IC_50_ of ~2 μM, while highly resistant to normal human lung fibroblasts (WI38) with IC_50_ of 48.5 μM (24 fold). 

The synthesis of **NMK-BH3** was achieved as shown in Scheme 7 [80]. A solution of indole-2-carboxylic acid (**42**) was refluxed in ethanol with a catalytic amount of dilute sulfuric acid, to obtain the corresponding ethyl ester intermediate, which was treated with hydrazine hydrate to obtain the intermediate **43**. On the other hand, the preparation of the indole-3-carboxaldehyde **45**, via treatment of the methoxy indole **44** with phosphorous oxychloride and anhydrous DMF. Finally, condensation of hydrazide intermediate **43** and indole-3-carboxaldehyde **45** in the presence of a catalytic amount of glacial acetic acid, and the mixture was refluxed at 80 °C for 5 h. The resulting mixture was then cooled to collect the desired target (**NMK-BH3**).

### 2.9. Inhibition of DNA-Topoisomerases

**Scheme 8:**
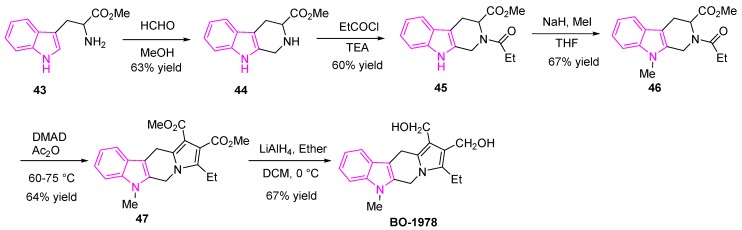
Synthesis of BO-1978 adapted from the references [81,82].

**Figure 8:**
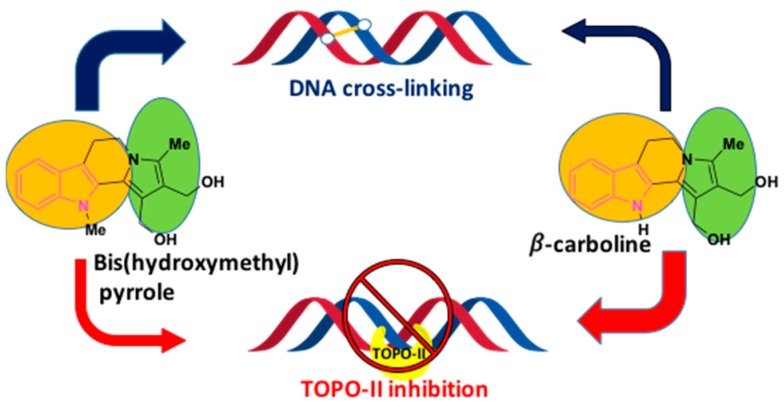
Biological significance of bis(hydroxymethyl)pyrrole and β-carboline.

Owing to the drug resistance and acute toxicity associated with lung cancer treatment, the combination of two or more pharmacophores has become an attractive strategy in drug discovery and has evolved as an effective tool in treating lung cancer patients. To overcome the multi-drug resistance in NSCLC patients, Chen et al. adapted the above strategy and developed hybrid molecules to improve drug efficacy. They designed and synthesized a series of indolizino[6,7-*b*]indoles, incorporating two pharmacophores: β-carboline group and a bis(hydroxymethyl)pyrrole group with topoisomerase (Topo) I/II inhibition and DNA cross-linking abilities respectively [81,82] (Figure 8). The hybrid derivative (**BO-1978**) was found to be effective in the suppression of lung adenocarcinoma A549 cells through the inhibition of Topo I/II and the induction of DNA cross-links resulting in DNA damage and a resulting cell cycle arrest. The hybrid derivative (**BO-1978**) was synthesized as shown in Scheme 8 [81,82].

The tetrahydro-β-carboline **44** was synthesized by adding formaldehyde to L-tryptophan methyl ester hydrochloride (**43**). The reaction of compound **44** with propionyl chloride in triethylamine provided the amide (**45**). The nitrogen of the indole intermediate (**45**) was methylated using sodium hydride and methyl iodide to generate the compound **46**. The reaction of **46** with dimethyl acetylenedicarboxylate (DMAD) in acetic anhydride yielded the diester **47,** which was further reduced with lithium aluminum hydride to afford the desired compound (**BO-1978**) (Scheme 8).

The hybrid derivative compound (**BO-1978**) showed the synergistic effect of both the pharmacophores, which incorporates the anti-proliferative activity and Topo I and Topo II inhibition of β-carboline and the potent anti-tumor activity of bis-hydroxy-methylpyrrole, which is known to induce the DNA cross-linking (Figure 8).

**Figure 9:**
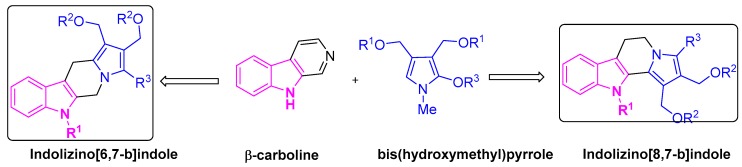
Design of indolizino[6,7-b]indole and indolizino[8,7-b]indole derivatives.

In the same front, Chang et al. developed a series of novel indolizino[8,7-b]indole hybrids (Figure 9) [83].

**Scheme 9:**
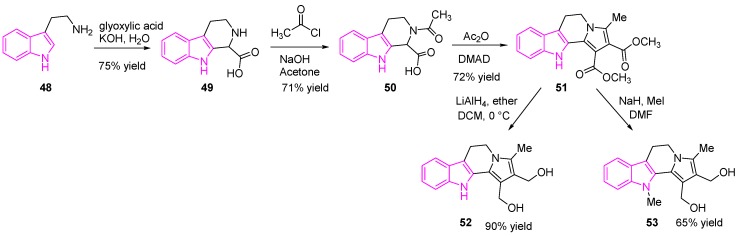
Synthesis of compounds **52** and **53,** adapted from the reference [83].

As shown in Figure 8, the substituent on the indole nitrogen is found to play a key role in determining the activity of the hybrid molecule. The presence of –Me group (**53**) makes them potent DNA cross-linking agents, whereas –NH derivatives (**52**) are potent TOPO-II inhibitors. The resulting compounds were most sensitive to SCLC (H526) cells both in vitro and in vivo studies. One of the compounds **52**, was found to have potent Topo II inhibitory activity with significant tumor suppression in nude mice bearing SCLC H526 xenografts (Scheme 9). Compound **52** was shown to be more potent than cisplatin and etoposide and possessed the comparable potency of irinotecan [83].

The synthesis of compounds **52** and **53** is shown in Scheme 9 [83]. Briefly, the reaction of tryptamine hydrochloride (**48**) with an aqueous solution of glyoxalic acid and potassium hydroxide generated the tetrahydro-β-carboline intermediate (**49**). Compound **50** was prepared by the addition of acetyl chloride to **49**, then DMAD was added to compound **50** in acetic anhydride generated the tetracyclic diesters (**51**). Finally, the reduction of **51** with lithium aluminum hydride afforded the desired tetracyclic diol (**52**), while the treatment of intermediate **51** with sodium hydride and methyl iodide resulted in desired N-methyl-tetracyclic diol (**53**).

**Scheme 10:**
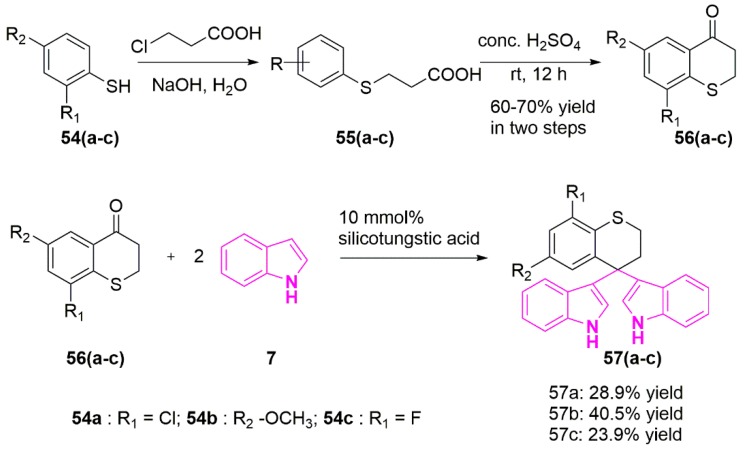
Synthesis of compounds **57**(**a–c**) adapted from reference [84].

In an attempt to develop effective Topo-II inhibitors as potent anti-cancer agents, Song et al. synthesized a novel series of bisindolylalkanes analogs such as 3,3′-(thiochroman-4,4-diyl)bis(1H-indole) and tested against a variety of human cancer cell lines (Scheme 10). Notable among them, compounds **57(a-c**) were found to be potent against A549 cells with IC_50_ values of ~9 µg/mL [84].

The synthetic scheme of compounds **57**(**a–c**) was shown in Scheme 10 [84]. The first step involved the condensation of substituted benzene thiols **54**(**a–c**) with 3-chloropropanoic acid and sodium hydroxide in water, followed by treatment of corresponding intermediate **55**(**a–c**) with concentrated sulfuric acid to obtain the intermediates **56**(**a–c**). A mixture of indole (**7**) and substituted thiochroman-4-ones **56**(**a–c**), in the presence of silicotungstic acid was refluxed in ethanol yielded the desired compounds (**57**(**a–c**)).

**Scheme 11:**
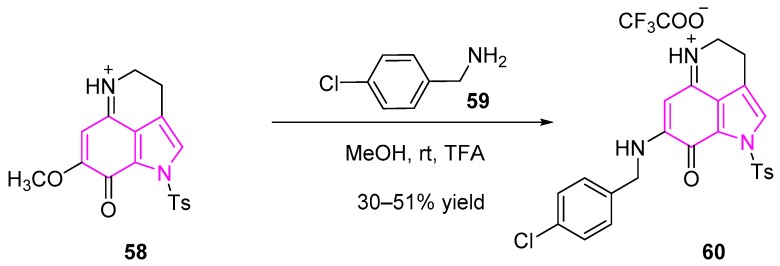
Synthesis of compound **60** adapted from reference [86].

Makaluvamine is an indole based alkaloid metabolite isolated from a marine sponge Zyzzya. This compound showed significant cytotoxic activity and DNA topoisomerase II inhibition [85]. Nadkarni et al. synthesized a series of makaluvamine analogs and studied their biological activities against various human lung cancer cell lines (Scheme 11). One of the compounds (**60**) was found to be most potent against human lung cancer cell lines of both human adenocarcinoma (A549) and carcinoma (H1299) cells with IC_50_ of 0.3 and 0.58 µM, respectively. Further studies revealed that the compound induced both apoptosis and S-phase cell cycle arrest [86].

The synthesis of makaluvamine **60** is represented in Scheme 11 [86]. Compound **60** was synthesized in one-step synthesis from the tricyclic compound **58**. Condensation of compound **58** with 4-chloro-benzylamine (**59**) in anhydrous methanol followed by treatment with trifluoroacetic acid (TFA) afforded the desired makaluvamine compound (**60**).

### 2.10. Histone Deacetylase (HDAC) Inhibitors

**Figure 10:**
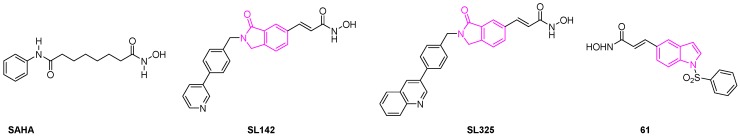
Structure of compounds suberoylanilide hydroxamic acid (SAHA), SL142, SL325, and **61**.

HDAC enzymes are involved in a variety of cellular processes such as cell proliferation, cell differentiation, and apoptosis [87]. Research studies show that the dysregulation of HDAC can lead to several types of tumor malignancies. HDAC inhibitors have, thus, evolved as a new class of anti-cancer agents because of their efficacy to arrest cell growth, cell differentiation, and cell death by apoptosis [88,89]. In this direction, Han et al. investigated the anti-cancer activity of A549 and H441 lung cancer cells using two novel cyclic amide-bearing hydroxamic acid-based HDAC inhibitors **SL142** and **SL325**. Their studies revealed that both derivatives showed more potent cell growth inhibition and cell death than the hydroxamic acid-based HDAC inhibitor suberoylanilide hydroxamic acid (SAHA) (Figure 10). Besides, these compounds can also induce significant caspase activity and subG_0_/G_1_ population in human epithelial (H441) and adenocarcinoma (A549) lung cancer cells, thereby demonstrating that they are more effective than **SAHA** [90,91].

A new class of 1-arylsulfonyl-5-N-hydroxyacrylamide indoles was reported by Lai et al., as potent histone deacetylase inhibitors. Further investigation revealed that the lead compound **61** demonstrated GI50 of 1 μM against the A549 cell lines. They demonstrated that the N-hydroxyacrylamide group located at the C-5 position of indole ring was more potent than other derivatives. When the group tested the efficacy of the compounds on a human xenograft model in nude mice bearing A549 lung cancer cell lines, compound **61** was found to be more effective than SAHA (Figure 10). The authors found that there was a 47% tumor growth delay (32% tumor growth inhibition) by oral administration with compound **61** daily at 200 mg/ kg showing its efficacy as a potential anti-lung cancer agent [92].

**Scheme 12:**
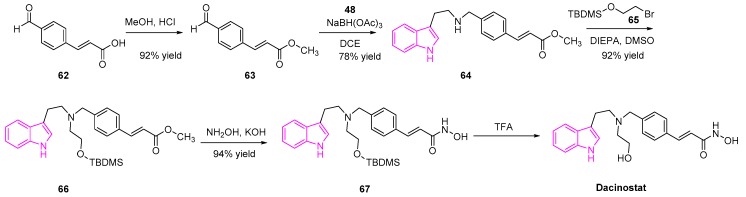
Synthesis of dacinostat adapted from the reference [98].

Dacinostat (NVPLAQ824) is an HDAC inhibitor introduced by Novartis in 2003 and is used as an anti-cancer agent (Scheme 12) [93]. Dacinostat was found to inhibit an HDAC enzyme isolated from H129 cell lysates with a potency of 32 nM and human lung cancer cells (H1299) with a potency of 150 nM, respectively. Besides, it was also found to be potent in the in vivo studies, in subject mice, against human lung carcinoma (A549 and HCT116) sub-cutaneous xenografts. Detailed studies of the mechanism of action showed that dacinostat acts on the A549 and HCT116 cells by G2-M arrest with a significant sub-G1 population, showing that the cells were subjected to apoptosis [94,95,96,97].

The synthesis of dacinostat is shown in the Scheme 12 [98]. Briefly, esterification of 4-formyl-cinnamic acid followed by reductive amination with tryptamine **48**, generated the condensed indole **64**. The resulting intermediate **64** was alkylated with 2-(tert-butyldimethylsilyloxy-ethylbromide (**65**) afforded the tertiary amine **66**. The methyl ester of **66** was transformed to hydroxamic acid **67**, followed by deprotection yielded the desired dacinostat (Scheme 12).

### 2.11. Miscellaneous Indole Derivatives in Anti-Lung Cancer Treatment

**Scheme 13:**
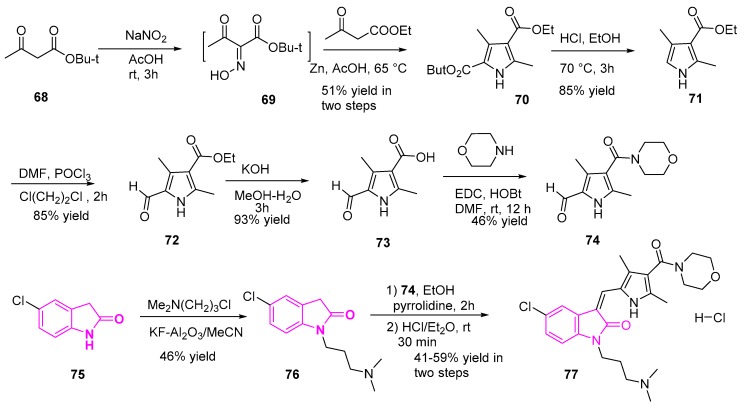
Synthesis of compound **77** adapted from references [99,100].

Lv et al. designed and synthesized a series of novel 1-(3-dimethyl-aminopropyl)indolin-2-one derivatives. Here the most active derivative was found to be more potent than sunitinib against NSCLC (A549) cells. These compounds were tested for their antiproliferative activity against lung adenocarcinoma (A549) cells and they found that all the compounds showed potent activity. Among all generated compounds, compound **77** (Scheme 13) proved to be most potent with IC_50_ of 1.10–1.47 µM, which is 1.8–6 fold higher than sunitinib [99].

A representation of the synthesis of the most potent compound **77** is shown in Scheme 13 [99,100]. Briefly, the reaction of tert-butyl acetoacetate **68** with sodium nitrite in acetic acid generated the oxime **69**. The resulting oxime (**69**) was reacted with ethyl acetoacetate and zinc powder in acetic acid yielded the pyrrole diesters (**70**). Ester hydrolysis of **70**, followed by monodecarboxylation reaction in the presence of conc. HCl and ethanol yielded the ethyl ester **71**. Then subsequent treatment of **71** with phosphorus oxychloride yielded the aldehyde **72**. The resulting intermediate (**72**) was hydrolyzed, followed by amide bond formation in the presence of morpholine and coupling reagents (EDC/HOBt) yielded compound **74**. On the other hand, compound **76** was obtained through the treatment of intermediate **75** with 3-chloro-N,N-dimethyl-propan-1-amine in the presence of KF-alumina. Finally, Knoevenagel condensation of the intermediates **76** and **74**, followed by HCl treatment generated the HCl salt of the desired target (**77**) (Scheme 13).

**Figure 11:**
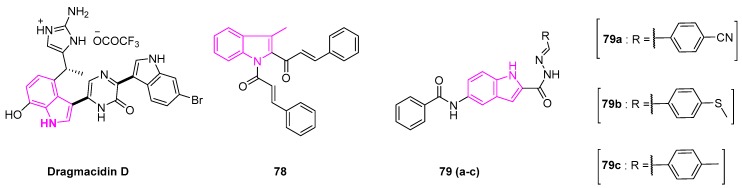
Structures of dragmacidin, compounds **78**, and **79**(**a**–**c**).

Dragmacidin is a cytotoxic bisalkaloid, isolated from a marine sponge Dragmacidin sp. This compound proved to be a potent antitumor agent. It showed IC_50_ value of 1–10 μg/mL against human lung cell lines (A549) in the in vitro studies (Figure 11) [101].

Recently, Zhao et al. reported a series of indolyl chalcones that can inhibit lung cancer cell growth. Of all the derivatives tested, they found compound **78** (Figure 11) to be effective in the induction of tumor apoptosis. It inhibited the growth of A549 cells by increasing the reactive oxygen species (ROS) levels and by activation of the The nuclear factor erythroid 2–related factor 2 (Nrf-2) pathway [102]. In the in vivo studies, compound **78** exhibited anti-growth activity of the tumor in an avian embryo model, showing the potential of the compound as the Nrf-2 activator in cancer therapy.

Hu et al. synthesized a series of indole derivatives with varying substituents at 2 and 5 positions. The resulting compounds exhibited excellent anti-proliferative activity on lung cancer cell lines (H460 and A549) (Figure 11). Notably, compound **79a** exhibited a high level of potency against A549 cells, with the IC_50_ value of 0.50 ± 0.06 μM, while compounds **79b** and **79c** exhibited IC_50_ values of 0.16 ± 0.05 μM and 0.48 ± 0.05 μM, respectively. Further detailed studies revealed that the compounds induced apoptosis by the inhibition of phosphorylation of Ser2 of the carboxyl-terminal domain of RNA polymerase II, where RNA polymerase II is essential for messenger RNA (mRNA) synthesis [103].

Huang et al. further identified a novel indole derivative, **SK228** that can effectively inhibit lung cancer cell lines by ROS production and induce cell death by apoptosis. To evaluate the potency of **SK228** against various human cancer cell lines, the authors treated the cells with varying concentrations of **SK228** for 48 h. The resulting IC_50_ values were found to be 3.4 and 0.3 μM for A549 and H1299 cells respectively [104,105]. Further investigation of the mechanism of action showed that **SK228** enhanced mitochondrial ROS production and inflicted DNA damage on cancer cells, ultimately leading to intrinsic mitochondria-dependent mitosis.

**Scheme 14:**
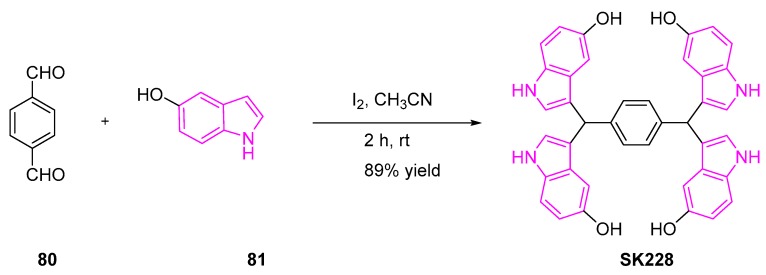
Synthesis of SK228 adapted from the reference [105].

**SK228** was synthesized in a single step, as shown in Scheme 14 [105]. A mixture of terephthalaldehyde (**80**) and 5-hydroxy-indole (**81**) in the presence of iodine afforded the desired tetraindoles product (**SK228**).

**Figure 12:**
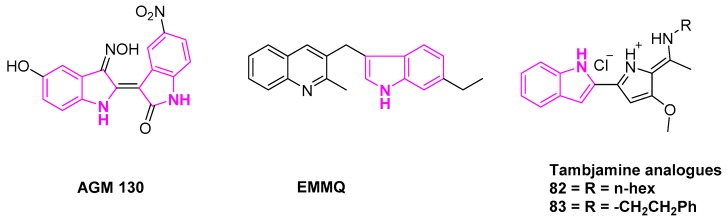
Structures of AGM130, EMMQ and tambjamine derivatives.

Kim et al. synthesized a novel indirubin-3′-oxime derivative **AGM130** with excellent anti-tumor activity and inhibition of cancer growth in A549 cells (Figure 12). Further investigation of the activity on A549 cells demonstrated that **AGM130** induced caspase-dependent apoptosis via the mitochondria-mediated intrinsic pathway [106].

Liu et al. reported a novel indolyl quinoline moiety 3-((7- ethyl-1H-indol-3-yl)-methyl)-2-methyl-quinoline (**EMMQ**) that can arrest cell-growth in NSCLC (A549 and H460) cells (Figure 12). Cell growth inhibition studies after 48 h of dose-dependent treatment indicated **EMMQ** IC_50_ values of 8 μM in both the A549 and H460 cell lines. This process involved the disruption of the mitochondrial membrane potential, DNA damage and ultimately apoptotic cell death [107].

To note, tambjamines are indole based marine alkaloids, known to be highly efficient transmembrane anion transporters in the model liposomes. They have widespread pharmacological applications (Figure 12). Studies have shown that these alkaloids can induce apoptosis in several cancer cell lines, rendering IC_50_ values in the micromolar range. Manresa et al. reported highly active indole based tambjamine derivatives, investigating their molecular mechanism of action. The results revealed that compounds **82** and **83** can induce apoptosis in lung cancer cells, in both in vitro and in vivo studies. Cytotoxicity is induced by the ROS activated stress kinase pathway. This occurs through the activation of P38 mitogen-activated protein kinase [108,109].

**Scheme 15:**
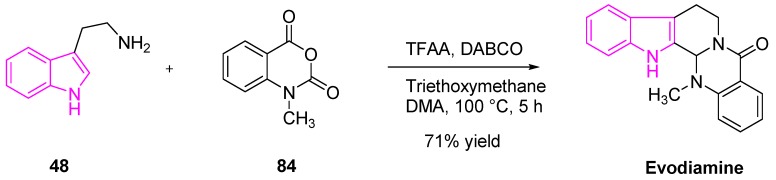
Synthesis of evodiamine adapted from the reference [114].

Evodiamine is an indole-containing alkaloid, isolated from a Chinese medicinal herb *Evodia rutaecarpa* (Wu-Chu-Yu). It has numerous pharmacological applications such as anti-inflammatory, anti-obesity, anti-viral, anti-tumor, and anti-nociceptive effects (Scheme 15). Studies have shown that evodiamine is capable of strong anti-tumor and cytotoxic activity against various human cancer cell lines (gastric, colon, liver, lung, and breast). Detailed mechanistic studies by Zou et al. revealed that evodiamine can exert the anti-tumor activity by the inhibition of Metadherin, which was identified as a novel oncogene that is overexpressed in many tumor malignancies [110]. Zou and his team demonstrated that the treatment of NSCLC cells (A549) with evodiamine results in an increased level of Bax expression. This is followed by a decrease of Bcl-2 and MTDH, which substantiated the apoptotic mechanism of action [111,112,113].

Evodiamine was synthesized as shown in Scheme 15 [114]. The reaction of tryptamine (**48**) with N-methylisatoic anhydride (**84**) in the presence of trifluoroacetic anhydride, 1,4-diazabicyclo[2.2. 2]octane (DABCO), triethoxymethane, and dimethylacetamide afforded the desired product of evodiamine (Scheme 15).

**Figure 13:**
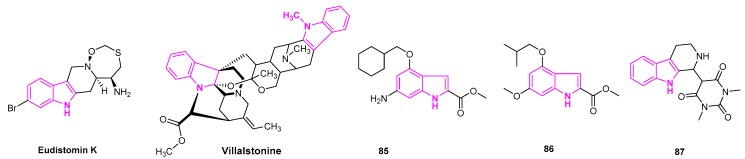
Structures of eudistomin K, villalstonine, compounds **85**, **86** and **87**.

An indole based alkaloid, Eudistomin K isolated from the Caribbean ascidian *Eudistoma olivaceum* was found to be potent against human lung cancer (A-549) cell lines. It is reported to inhibit the P-388 tumor cell line with IC_50_ of 0.01 µg/mL (Figure 13) [115].

Bisindolylalkanes comprise two monomeric indole alkaloid units, derived from several natural terrestrial and marine sources. They are known for their biological, pharmacological, and medicinal properties. Villalstonine is derived from the Alstonia species, which is commonly found in the tropical and subtropical regions. It was evaluated for its anti-cancer activity against human NSCLC cell lines, adenocarcinoma (MOR-P), and large cell carcinoma (COR-L23) cell lines. Villalstonine was demonstrated to be potent in cytotoxic activity with IC_50_ of 5 µM and 2.5 µM in MOR-P and COR-L23 cells, respectively (Figure 13) [116].

Ji et al. reported a novel class of indole-2-carboxylate derivatives, evaluating their anti-cancer activity. They found that some compounds exhibited excellent anti-proliferative activity against HepG2, A549, and MCF7 cells. Most importantly, compounds **85** and **86** showed more potency than etoposide and pyrroloquinoline quinone drugs with IC_50_ ranging from 3.4 to 24 µM (Figure 13). When compounds **85** and **86** were subjected to a Western blot analysis of poly ADP ribose polymerase (PARP), both compounds triggered ROS production and induced PARP cleavage in A549 cells. This study was done in a dose-dependent manner, indicating the induction of apoptosis. The results showed that these substances can be promising lead compounds for the development of anti-lung cancer drugs [117].

Skouta et al. designed and synthesized a series of tetrahydro-β-carbolines derivatives. These compounds were tested for their efficacy against a variety of human cancer cell lines, including the lung cancer cells (H1299). They found that derivatives of tetrahydro-ß-carboline and barbituric acid moieties with no spacer showed modest activity with LC50 of 32.8 µM in the H1299 cell lines. Those analogs with a carbon spacer were either weakly active or non-selective. Compound **87** was found to be the most potent than any of the derivatives. It was observed to induce caspase-independent cell death selectively in cancerous cells [118].

## 3. Conclusions

Overall, indole based compounds have emerged as a powerful probe for scientists in the ongoing battle against such life-threatening diseases, such as lung cancer. Significant advances in this area are evidenced in the number of indole-based compounds entering the pre-clinical and clinical stages. More advances are expected to meet the challenges associated with the accompanying chemotherapy. With a better understanding of the drug resistance mechanism in lung cancer patients, coupled with the multi-drug therapy advances offered by the indole-based derivatives, better and more promising strategies for combatting lung cancer can be expected in the near future.
